# Editorial: Germ cell development and reproductive aging

**DOI:** 10.3389/fcell.2022.1051539

**Published:** 2022-10-12

**Authors:** Miguel Angel Brieño-Enriquez, Francesca E. Duncan, Arjumand Ghazi, Michael Klutstein, Vittorio Sebastiano, Jessica Tyler

**Affiliations:** ^1^ Department of Obstetrics, Gynecology, and Reproductive Sciences, School of Medicine, Magee-Womens Research Institute, University of Pittsburgh, Pittsburgh, PA, United States; ^2^ Department of Obstetrics and Gynecology, Feinberg School of Medicine, Northwestern University, Chicago, IL, United States; ^3^ Departments of Pediatrics, Developmental Biology and Cell Biology, and Physiology, John G. Rangos Sr. Research Center, University of Pittsburgh School of Medicine, Pittsburgh, PA, United States; ^4^ Faculty of Dental Medicine, Institute of Biomedical and Oral Research, The Hebrew University of Jerusalem, Jerusalem, Israel; ^5^ Institute for Stem Cell Biology and Regenerative Medicine, Stanford University School of Medicine, Stanford, CA, United States; ^6^ Department of Obstetrics and Gynecology, Stanford University School of Medicine, Stanford, CA, United States; ^7^ Department of Pathology, Laboratory Medicine, Weill Cornell Medicine, New York, NY, United States

**Keywords:** aging, germ cells, oocyte, sperm, primordial germ cell, reproductive aging, ovary, testis

## Introduction

Aging and reproductive aging are influenced by environmental factors, stochastic aberrations in molecular interactions, and deterministic instructions encoded in our genome. Although no single process has been shown to be the main cause of aging, it appears that multiple processes contribute, leading to a loss of fitness and eventual death. Over the last 30 years, interest in the field of aging has exponentially increased since the pioneering discoveries of single gene mutations that double the lifespan of the nematode *Caenorhabditis elegans* (Kenyon et al., 1993). These genes and pathways have since been shown to be evolutionarily conserved and innumerable other genes and interventions that impact longevity across species have been discovered. However, these analyses have been predominantly focused on somatic tissues rather than the ovary and testis and how aging of reproductive tissues impacts somatic aging. Undoubtedly, one of the most important events in biology is the union of the sperm and egg—and their unique ability to give rise to an entirely new generation composed of billions of cells. Despite the importance and tremendous potential (immortal lineage) of these cells, the male and female gametes are not impervious to aging. In humans, prominent changes in social behavior and medical interventions have changed the landscape of reproduction, enabling a delay in childbearing and longer post-menopausal lifespan in women. All these factors have a profound impact on the quantity, quality, and ultimate reproductive potential of germ cells. Reproductive aging is not unique to mammals but affects many other organisms, including yeast, flies, and worms. Thus, understanding the fundamental mechanisms underlying reproductive aging is essential to develop new approaches to extend reproductive lifespan, which will ultimately impact overall health. The 20 articles in this Research Topic explore critical aspects of *germ cell development and reproductive aging*. They include 10 research articles, 8 reviews, and 2 mini reviews, covering from insects to humans.

## Overview

### From insects to human: Convergences and divergences in reproductive aging

Reproductive aging is not exclusive to humans or laboratory model organisms but also occurs in species in the wild including large mammals. Although many mechanisms that control aging are conserved among species, there are distinct traits exhibited by different species. Comizzoli and Ottinger review the conserved mechanisms of reproductive aging and highlight the value of comparative studies addressing aging issues in conservation breeding as well as human reproductive medicine. Many of these conserved mechanisms are well known, such as depletion of the ovarian reserve or decline in testicular function, as well as phenomena observed in social insects where organismal senescence regulates aging rather than germ cell senescence. They also discuss how corals, birds, tardigrades, fish, reptiles, social insects like ants and bees and naked mole-rats do not show a decline in fertility with age and can provide knowledge that can lead to potential interventions to improve fertility in aged humans (Nisbet et al., 1999; Heinze and Schrempf, 2008; Tsujimoto et al., 2016; Place et al., 2021; Buffenstein et al., 2022).

The major determinants of reproductive aging are defects in germ cell expansion during development or premature depletion of the germline stem and progenitor pools (Antebi, 2013). On this subject, Tolkin and Hubbard review recent advances in our understanding of the role of depletion of the germline stem and progenitor cell pools in *C. elegans.* Their review dissects the role of specific molecular pathways, such as Notch and insulin/IGF like receptors, in the decline of germ cells with age. In their review, Wu et al., explore the mechanisms of vitellogenesis in insects, a critical process for egg production and embryonic development and that addresses the importance of lipids and specific germ cell proteins across species. Also, using *C. elegans* as a model, Scharf et al., performed a detailed analysis of the mechanisms involved in reproductive aging and chemical interventions that can help delay aging in nematodes. Notably, Scharf et al., also describe how reproductive aging influences birth rate affecting population dynamics. In both reviews, the authors discuss new avenues of research that can improve the effects of aging in worms and how it can be extrapolated to humans. An example of potential translation to humans is presented by Choi et al., who show that oleic acid-mediated increased reproduction can be dissociated from somatic longevity, reducing the energetic cost of reproduction.

### Aging the proteome: Dynamics of mRNA translation and proteostasis

Regulation of mRNA translation is critical for the proper function of the cell. In the case of germ cells, there is accumulating evidence to suggest that changes in mRNA translation can contribute to infertility and reproductive aging in organisms from insects to humans. For this Research Topic, Mercer et al., review the dynamic regulation of mRNA translation and ribosome biogenesis in germ cells of flies, worms, and mice. The authors evaluate the conserved functions between species, their differences, and suggest potential new lines of research in this field. Changes in mRNA translation and ribosomal biology can directly affect proteostasis. Cafe et al., review the role of proteostasis in the maintenance of the germline and in reproductive health. In their review, the authors analyze proteostasis in the context of both somatic and germ cell aging, and how disruption of proteostasis leads to defects in gametes, embryogenesis, and lifespan. Intracellular communication by extracellular vesicles is key for many physiological and pathological processes and is mediated *via* cargo including nucleic acids (DNA, RNA, microRNAs, and long-non-coding RNAs) proteins and enzymes (Yanez-Mo et al., 2015). Liu et al., performed a review of the roles of extracellular vesicles in aging and their implications in reproductive diseases.

### Aging is for all: Impact of aging on male germ cells

Primordial germ cells (PGCs) and spermatogonial germ cells are critical for reproduction and disruption of their proliferation or migration results in fertility defects. Aging also impacts stem cells and old spermatogonial stem cells have reduced function and ability to generate sperm (Liao et al., 2021). Defects at early stages of germ cell development are not unique to females. Zhou et al., employ a unique model, the viviparous fish black rockfish, and use different molecular approaches to trace the origin and migration of the PGCs in fish. Another powerful approach to study germ cell development is described by Xu et al., where they use mass spectrophotometry to reveal differences in protein abundance and phosphorylation in the sperm of Yorkshire and Duroc pigs and the authors discuss the relationship between pig reproductive efficiency and fertility. Also related to male germ cell development, Zhou et al., shows that proliferation and apoptosis in human spermatogonial stem cells (SSCs) is regulated by TCF3, a member of the E-protein family of helix-loop-helix transcription factors (Slattery et al., 2008). They find that the effect of knocking down TCF3 *in vitro* produces phenotypes reminiscent of TCF3 deficient patients with non-obstructive azoospermia.

### Too many avenues with a common end: Reproductive aging in females

Female reproductive aging is characterized by a loss of follicles, the functional units of the ovary consisting of oocytes surrounded by companion granulosa cells. The quality of the oocytes in the ovary also deteriorates with age. Reproductive aging is associated with adverse reproductive outcomes, including infertility, miscarriages, and birth defects (Duncan and Gerton, 2018; Gruhn et al., 2019). Moreover, reproductive aging is associated with a decline in estrogen, which is produced by growing follicles. Estrogen regulates cardiovascular, brain, immune, bone, and reproductive tissue function (Satirapod et al., 2020). In fact, menopause accelerates aging (El Khoudary et al., 2020). In this special Research Topic, 8 papers focus exclusively on different aspects of female reproductive aging. Liu et al., evaluate the role of the ten-eleven translocation (Tet) enzymes in the establishment of the ovarian reserve. They show that in the absence of Tet-1, young mice have a severe reduction in the number of follicles and become infertile by middle age. These mutants also present disruption of organelle fission, ubiquitination, autophagy, and upregulation of expression from transposable elements such as Line1. Aging not only impacts the pool of germline stem/progenitor cells, it also affects later stages of gametogenesis. One clear example is presented by Raices et al., where they demonstrate that the capacity to repair DNA damage during meiosis in the germ cell is reduced with age. Raices et al., evaluated the recruitment of the proteins RPA-1 and RAD51 that are required for homologous recombination, showing that the dynamics of early meiotic DNA damage and repair are compromised in older mothers, increasing the risk of aneuploidy and low oocyte quality.

### Beyond the germ cells: Ovarian stroma, inflammation and hormones during aging

Depletion of ovarian reserve is not only regulated by the intrinsic characteristics of the germ cells, but also local environment such as the health of the stroma (Amargant et al., 2020; Umehara et al., 2022), inflammation (Foley et al., 2021; Navarro-Pando et al., 2021) and hormonal balance. Non-infectious chronic and mild inflammation is a hallmark of aging. Products of this inflammation can be detected by inflammasomes (complexes of multiple proteins that induce sterile inflammation). Regulation of inflammasomes is highly controlled by different proteins including NACHT, LRR and PYD domains-containing protein 3 (NLRP3). Liberos et al., using *Nlrp3* knockout mice, show that ablation of this gene diminished the pro-inflammatory cytokines and macrophage infiltration leading to a larger ovarian reserve. However, aging is not the only cause of inflammation in the ovary. In fact, in this Research Topic Knapik et al., review the role of T cells in ovarian physiology and disease from polycystic ovary syndrome to ovarian cancer, and how each one of these pathologies can lead to reproductive aging and infertility.

Regulation of the reproductive cycle in females is tightly regulated by hormones. In this Research Topic Björkgren et al., using mice as a model, ablate the α/β hydrolase domain-containing protein 2 (*Abhd2*). Mutant mice showed disruption of the estrus cycle rhythm. Abnormal cycles were accompanied by a phenotype that mimics the histology observed in polycystic ovaries, revealing the relevance of this gene as a non-genomic steroid regulator of the female reproductive cycle and a potential target for therapy in humans.

### Protecting the quality of the egg: Is there a right time to cryopreserve the oocyte?

The quality of the oocyte is critical for fertility. In this Research Topic Beverley et al., review the impact of cohesin variants and mutations in reproductive aging, disease, and overall health. Aging is directly associated with loss of the meiosis-specific cohesion, that leads to premature segregation of sister chromatids, aneuploidy, and miscarriages. One avenue to combat oocyte aging is cryopreservation. This technique is an important option for patients that need to go through medical treatments such as chemotherapy and radiotherapy but also for females planning to delay motherhood. However, not all oocytes are equally capable of *in vitro* maturation or fertilization. Studies in this Research Topic from Kusuhara et al., and Karavani et al., in both mice and humans (respectively) identify optimal time windows for retrieving mouse and human oocytes for cryopreservation. Their findings demonstrate that younger is not always better and that oocytes harvested during the pubertal transition have a reduced competence relative to reproductively adult mice. Sirait et al., review the impact of the characteristics of the cumulus-oocyte complex, such as of the nuclear maturation, apoptosis, extracellular matrix remodeling and steroids metabolism, as biomarkers for *in vitro* fertilization outcomes.

## Conclusion

Over the past several years, significant progress has been made in our understanding of the biological functions and key events during reproductive aging. Despite the advances described above, many aspects of the biological processes involved, and treatments to improve them, remain poorly understood. Therefore, mechanistic studies are necessary to identify the molecular networks that drive reproductive aging. We need to better understand the role of other cells beyond the gametes, and the role played by the extracellular matrix, hormones, non-coding RNAs, proteostasis, and epigenetics in reproductive aging. Future identification of the molecular basis of dynamic transcriptional regulation and protein post-translational modifications in germ cells and reproductive aging remains a crucial endeavor. Understanding molecular mechanisms will allow us to develop new technologies and therapies to improve reproductive health, reproductive lifespan and organismal healthspan.
